# Synovial Fluid Cell Proteomic Analysis Identifies Upregulation of Alpha-Taxilin Proteins in Rheumatoid Arthritis: A Potential Prognostic Marker

**DOI:** 10.1155/2020/4897983

**Published:** 2020-04-23

**Authors:** Ashish Sarkar, Shivani Sharma, Prachi Agnihotri, Tanmoy Sarkar, Pooja Kumari, Rajesh Malhotra, Barun Datta, Vijay Kumar, Sagarika Biswas

**Affiliations:** ^1^Council of Industrial Research (CSIR)-Institute of Genomics and Integrative Biology, Mall Road, Delhi University Campus, 110007, Delhi, India; ^2^All India Institute of Medical Sciences, Ansari Nagar, New Delhi 110029, India; ^3^Army Hospital Research And Referral (RR Hospital), Dhaula Kuan, New Delhi, India

## Abstract

Rheumatoid arthritis (RA) is a chronic autoimmune inflammatory disease affecting the joints and surrounding tissue. Identification of novel proteins associated with the progression of a disease is a prerequisite for understanding the pathogenesis of RA. The present study was undertaken to identify the potential biomarkers from a less explored biological sample such as synovial fluid (SF) cells which is specific for RA and to analyze their functional aspects using proteomic approach. Two-dimensional gel electrophoresis (2-DE) was performed using synovial fluid cells of RA and osteoarthritis (OA) patients, and 7 differentially expressed proteins were identified using matrix-assisted laser desorption/ionization time-of-flight mass spectrometry (MALDI-TOF MS/MS). Αlpha-Taxilin (*α*-Taxilin) has been found as one of the novel, significantly up regulated protein in RA. It has been validated in the synovium, synovial fluid (SF), SF cells, and plasma samples by Western blot, enzyme-linked immunosorbent assay (ELISA), fluorescence-activated cell sorting (FACS), immunohistochemistry (IHC), and real-time PCR. The identification of autoantibody against *α*-Taxilin and in silico studies has further helped us to understand its involvement in disease mechanism. The present study will therefore provide knowledge towards the etiology of RA that pave the way for suitable prognostic marker identification along with other clinical parameters.

## 1. Introduction

Rheumatoid arthritis (RA) is the most common chronic, systemic inflammatory autoimmune disease affecting around 1% of the population worldwide [1, 2]. It is often accompanied by systemic manifestation such as anemia, fatigue, and osteoporosis [3]. In spite of many efforts, the etiology of RA is still not clear [4, 5]. As the understanding about the disease is very limited, symptomatic treatment is mainly by the use of disease-modifying antirheumatic drugs (DMARDs), biologics, steroids, or combinations thereof [6, 7].

RA is believed to be mediated by an immune complex that does not clarify the acute inflammatory features of the disease [8]. Tumor Necrosis Factor (TNF), IL-6, anticyclic citrullinated peptide (anti-CCP), rheumatoid factor (RF), and antimannose binding lectin [9] (anti-MBL) are helpful in diagnosing the disease to a certain extent. However, these antigens are not specific whether they initiate autoimmune reactions, thus making the diagnosis critical [8, 10]. As the early stage of RA is characterized by nonspecific clinical symptoms, a delayed diagnosis leads to irreversible joint damage [11, 12]. Thus, there is an urgent and strong need for novel and more precise biomarkers with higher sensitivity and specificity for early diagnosis of disease. The study can therefore complement the conventional measures which may provide a more efficient marker to diagnose the disease for timely initiation of available therapies. Since the specific site of inflammation plays a vital role in the disease progression, synovial fluid cell (SF cell) has been used as primary source to study the differentially expressed proteome profile. Additionally, synovium and plasma samples were also used to strengthen our findings.

We used two-dimensional gel electrophoresis (2-DE) followed by matrix-assisted laser desorption/ionization time-of-flight mass spectrometry (MALDI-TOF MS/MS) [13] to study the differential proteome profile of RASF compared to OASF cells. OA SF cells were used as control SF cells since OA is an old age inflammatory joint disease but lacks autoimmune response [4]. OA is characterised by accumulation of synovial fluid and inflammation; thus, synovium and synovial fluid samples from OA patients have been widely used as control for RA study [14, 15]. Following the study, a total of 17 differentially expressed protein spots were observed. Among them, 7 spots have been identified successfully by mass spectrometry analysis. “TXLNA” or “Alpha-Taxilin/*α*-Taxilin” also known as interlukin-14 (IL-14) [16] or “High molecular growth factor” was found to be the most significantly upregulated protein in RA. Alpha-Taxilin belongs to the Taxilin family, has three members which include Alpha- (*α*-), Beta- (*β*-), and Gamma- (*γ*-) Taxilin. While *α*-Taxilin and *γ*-Taxilin are globally expressed, *β*-Taxilin (MDP77) is only expressed in the heart and skeletal muscles [16, 17]. *α*-Taxilin binds to proteins of the syntaxin family that are localized on the plasma membrane [18, 19]. The role of *α*-Taxilin has been reported earlier in exocytosis and cytokine activity which leads to inflammation via B-cell activation [20]; interestingly, literature support that an increased level of *α*-Taxilin has been found responsible for development of autoimmunity in transgenic mice [21], thus making our finding more relevant and important for understanding the etiology of RA.

## 2. Material and Methods

### 2.1. Sample Collection

Whole blood samples (RA = 100 (Age 50 y ± 5, male+female, Disease activity score (DAS) 28 = 6 ± 0.5), OA = 100 (Age 50 y ± 5, male+female) were collected hygienically in the EDTA-coated vacutainer (Ptech) from All India Institute of Medical Sciences (AIIMS, Ansari Nagar, New Delhi, India) and Army Hospital Research and Referral Hospital (Dhaula Kuan, New Delhi, India). All enrolled participant patients had fulfilled the 2010 American College of Rheumatology criteria for RA and OA diagnosis, having radiological evidence and clinical history (detail clinical parameter is given in “Supplementary table 1”). Healthy volunteers (HC = 64) also requited in this study having no radiographic evidence of joint degradation and other related clinical history. Synovial fluid (6 ml to 8 ml) were collected from RA (*n* = 16) and OA (*n* = 16) patients, and the synovium was collected after biopsy from RA (*n* = 6) and OA (*n* = 6), respectively. All the patients and healthy group provided signed written informed consent for these studies and were explained of all the associated risks before sample collection.

### 2.2. Isolation and Extraction of Proteins from Synovial Fluid Cells

Patient's SF (≈ 8 ml) was centrifuged at 4000 × g for 5 min in a swinging bucket rotor at 4°C. Cells were collected and washed 3 times with phosphate-buffered saline (PBS) at 300 × g for 5 min and incubated for 30 min at 4°C in SF cell lysis buffer (25 mM Tris, 1% Nonidet P-40, 150 mM Sodium Chloride (NaCl), 1.5 mM Ethylenediaminetetraacetic acid (EDTA), 0.5% Sodium dodecyl sulfate (SDS), 1 mM phenylmethane sulfonyl fluoride (PMSF), and 1% *v*/*v* Protease Inhibitor cocktail (PI cocktail) followed by sonication at 20% amplitude for 5 min. The cell lysate was then centrifuged at 15000 × g for 30 min, and the supernatant was collected for further experiments.

### 2.3. Two-Dimensional Gel Electrophoresis (2-DE)

Blood plasma samples (RA = 12, OA = 12, 50 y ± 5, male : female, 1 : 1) were pooled and quantified by the Bradford assay [22]. Three sets of 2-DE gels were run by using a pooled plasma sample from RA and OA after small modification [23]. Briefly, 150 *μ*g protein was added to the rehydration buffer, loaded on immobilized pH gradient (IPG) strips (17 cm, pH 4–7 (Bio-Rad Laboratories), and was separated in the first dimension by isoelectric focusing (IEF) followed by the second dimension using sodium dodecyl sulfate-polyacrylamide gel electrophoresis [23] (SDS-PAGE).

### 2.4. Silver Staining

After 2-DE, gels were kept in fixative (50% methanol and 12% acetic acid) for an hour [24], followed by washing with 50% ethanol and 30% ethanol for 30 min each. Gels were then sensitized with 0.2% Na_2_S_2_O_3_ for 1 min, washed with Milli-Q thrice, and stained with 1 M AgNO_3_ and 700 *μ*l/lit formaldehyde (HCHO) solution for 20 min followed by the development of gels using a developer consisting Na_2_CO_3_(60 gm/lit), HCHO (500 *μ*l/lit), and Na_2_S_2_O_3_ (25 mm) [23]. The reactions were then stopped by adding 6% acetic acid.

### 2.5. In-Gel Digestion of Proteins and Preparation for MALDI-TOF MS/MS Analysis

For identification, differentially expressed protein spots were digested with trypsin gold (Promega, USA) as earlier [4]. Briefly, protein spots were excised into 1 mm cubes, washed with Milli Q, destained with 30 mM potassium ferricyanide and 100 mM sodium thiosulfate (1 : 1) at RT, and dehydrated by incubation with 1 : 1 acetonitrile (ACN)/water. Followed by washing with ACN, gel pieces were rehydrated (10 mM NH_4_HCO_3_ equal volume of ACN), vacuum dried, and digested at 37°C for 18 h with 20 *μ*l of trypsin (20 ng/*μ*l, Trypsin Promega). Peptides were extracted by varying concentrations of ACN and trifluoroacetic acid (TFA, Sigma-Aldrich, St. Louis, MO, USA). Peptide concentrate (1 *μ*l) and sinapinic acid (1 *μ*l) were mixed and air dried and run using MALDI-TOF MS/MS (Applied Biosystems, Life Technologies, USA) as earlier [4]. MS/MS spectra were procured in reflector positive mode over the mass scope of 10,000-20,000 Da, and proteins having more than or equal to 2 unique peptides were considered.

### 2.6. Western Blot

Western blot was carried out as earlier [23] with little modification. Proteins (40 *μ*g) were separated by running in 12% SDS gel and transferred to nitrocellulose(NC) membrane (Millipore, USA) at 20 V for 20 min using a semidry Western blot unit (Bio-Rad) followed by overnight blocking (5% BSA in 1x PBS) at 4°C. After washing 3 times with PBST (0.05% Tween 20 in 1x PBS), the NC membrane was incubated for 2 h with (dilution 1 : 5000) anti-*α*-Taxilin (sc 271783). The membrane was washed again and incubated with (dilution 1 : 7000) horse raddish peroxide- (HRP-) conjugated anti-mouse for 1 h. The blots were developed by enhanced chemiluminescence (ECL) reagent (G-Biosciences).

### 2.7. Autoantibody Detection

*α*-Taxilin protein was immunoprecipitated using catch and release v2.0 (Millipore, Cat 17-500) and separated on 10% SDS-PAGE. The proteins were transferred on the NC membrane using the semidry western method (Bio-Rad). The presence of autoantibody against *α*-Taxilin in plasma was checked by incubating the blot with RA, OA, and healthy plasma (1 : 500) individually as a source of primary antibodies followed by incubation with anti-human HRP-conjugated secondary antibody (dilution 1 : 5000) and developed by ECL substrate.

### 2.8. Enzyme-Linked Immunosorbent Assay (ELISA)

ELISA was performed using RA (*n* = 100) and OA (*n* = 100) plasma samples and healthy (*n* = 62) individual plasma samples along with RASF (*n* = 16) and OASF (*n* = 16) samples. ELISA microtiter plates (Nunc, USA) were coated with 100 *μ*l diluted sample (plasma/SF; dilution 1 : 200) and incubated at 4°C overnight. After washing, wells were blocked with 1% BSA for 1 h at RT followed by 2 h incubation with 100 *μ*l diluted (1 : 2000) anti-*α*-Taxilin (Santacruz, USA) at RT. Plates were then washed and incubated with 100 *μ*l HRP-conjugated secondary anti-mouse antibody (dilution 1 : 1000) (Jackson, USA) for 1 h at RT. The reactions were then developed with orthophenylene diamine (1 mg/ml) substrate for 15 min, terminated by adding 50 *μ*l of 3 N·H_2_SO_4_. The absorbance was measured at 492 nm (SpectraMax Plus 384) [25].

### 2.9. Fluorescence-Activated Cell Sorting (FACS)

Fluorescence-activated cell sorting (FACS) was carried out using leukocytes and SF cells. Approximately 2 million cells were taken from each group and fixed with fixative (Cytoperm BD) for 15 min and then washed and blocked with 1.5% BSA for 30 min in their respective vials. After washing, cells were incubated for 2 h with 200 *μ*l diluted (1 : 2000) anti-*α*-Taxilin antibody followed by incubation with 200 *μ*l anti-mouse (Alexa Fluor 467) secondary antibody (1 : 1000) for 30 min. Cells were washed again with PBS and resuspended in 500 *μ*l of PBS, and 10000 cells were acquired in each run using FACS caliber (BD Biosciences) and analysed.

### 2.10. Immunohistochemistry (IHC)

Tissue samples RA (*n* = 6) and OA (*n* = 6) were fixed in 10% formalin for 1 h, paraffin-embedded, and cut into desired thickness (5 *μ*m) using a microtome. Samples were then submerged in citrate buffer (pH 6.0), incubated at 40°C for 2 min periods repeatedly for three times, and then finally kept for 20 min in a microwave. The tissue sections were then equilibrated at RT in the buffer (3% formaldehyde) and rinsed with Milli-Q water. The anti-*α*-Taxilin antibody was then added to the sections at a 1 : 400 dilution and incubated overnight at 4°C. Anti-mouse HRP conjugated was used as a secondary antibody (1 : 200 dilutions) and incubated for 2 h. The sections were counterstained with hematoxylin for 20 s followed by incubation with freshly prepared 3, 3′-diaminobenzidine (DAB, Sigma-Aldrich, USA) till the section developed. The slides were then mounted with a cover slip and observed under a microscope [26].

### 2.11. RNA Extraction and Real-Time PCR

Total cellular RNA from SF cells were extracted using Trizol (Gene Mark), following the manufacturer's instructions, and the cDNA was obtained by reverse transcriptase (Fermentas, source MULV). Quantitative real-time PCR was performed using ABI SYBR green PCR master mix and amplified by (Applied Biosystem StemOnePlus™) real-time PCR thermal cycling block. The primers for *α*-Taxilin were as follows: FP 5′-GGTTTGGGGAAGGAGATCACG, RP 5′-GGAGCTTCATCTGCTTCTGTG.

The fold change of mRNA was calculated using the comparative Delta threshold cycle (*∆*Ct) method with 18S as the loading control. All reactions were performed in triplicate for each sample.

### 2.12. String Pathway Analysis and Interaction Study

Online tool “STRING” pathway has been used to find out the interacting partner of *α*-Taxilin. Apart from collecting and reassessing available experimental data from database on protein-protein interactions, imported known biological pathways from curated database interaction predictions were made [27] by this tool.

### 2.13. Statistical Analysis and Software

The patient DAS-28 score was calculated using a freely available online DAS-28 calculator (Rheumakit) by providing the patient clinical parameter such as tender joint, swollen joints, ESR, and overall health status. Densitometric analysis was carried out using the “Image Lab v 3.00” (Bio-Rad Laboratories) analysis tool for all Western blots. A bar graph for all Western analysis was plotted using mean adjusted densitometric values obtained in Image Lab analysis with the help of Microsoft Excel (Microsoft Corporation). A dot plot for ELISA obtained by putting absolute absorbance for each group in “GraphPad Prism 7” and *p* values were obtained. The standard deviation was calculated for replicates and plotted on the bar graph. Data having a *p* value less than 0.05 was considered significant.

## 3. Results

### 3.1. Identification of Differentially Expressed Proteins by Two-Dimensional Gel Electrophoresis (2-DE)

**Figure 1:**
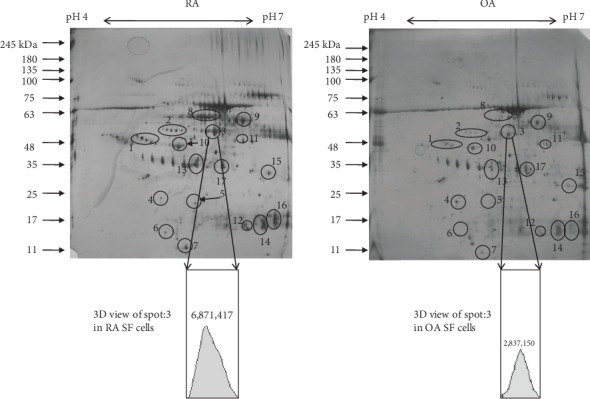
Differential proteome profile. Protein profile of synovial fluid cells of RA and OA showing differentially expressed protein spots in synovial fluid cell lysate using 2D-SDS PAGE gel. 150 *μ*g of protein sample was loaded in IPG (pH 4-7) strip for 2D SDS-PAGE followed by silver staining.

2-DE was carried out to compare the protein profile of RASF and OASF cells. MALDI-TOF MS/MS analysis successfully identified 7 proteins out of 17 marked differentially expressed protein spots from 2-DE (Figure 1). Among these identified spots, regulatory protein E2 (spot 1), shikimate kinase (spot 2), *α*-Taxilin (spot 3), protein kinase A type 1a regulatory subunit (spot 5), recombinant signal binding protein (spot 6), and putative enoyl (spot 15) were found to be upregulated in RA while “chain A structure of Hop Tpr2a domain in complex”(spot 4), a stress-induced phosphoprotein, was observed to be downregulated in RASF cells (Table 1). After performing densitometric analysis of the 2-DE gel image, we found that *α*-Taxilin is one of the significantly upregulated (2.4) RASF cells compared to OASF cells.

### 3.2. Validation of *α*-Taxilin by Western Blot

**Figure 2:**
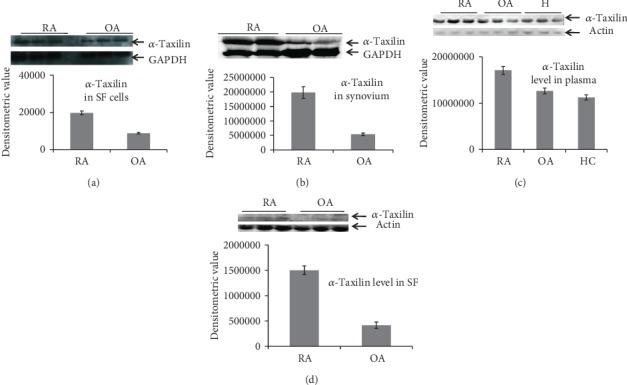
Validation by Western blot. Graph showing mean densitometric values obtained after Western blot analysis in different biological samples. (a) Expression level of *α*-Taxilin in synovial fluid cells of RA and OA. (b) Expression level of in the synovium of RA and OA. (c) Expression level of *α*-Taxilin in blood plasma of RA, OA, and HC. (d) Expression level of *α*-Taxilin in synovial fluid of RA and OA.

Western blot analysis of RASF cells showed 2.22 fold and 3.6 fold up regulation of *α*-Taxilin as compared to OASF cells and OASF, respectively (Figures 2(a) and 2(d)). Similarly, the expression of *α*-Taxilin was found to be 1.68-fold higher in RA synovium as compared to OA synovium (Figure 2(b)). Furthermore, the expression was also compared in plasma samples of RA, OA, and HC (Figure 2(c)). Expression of *α*-Taxilin has been found to increase by 1.52-fold and 1.35-fold in RA plasma compared to HC plasma and OA plasma (*p* ≤ 0.048), respectively.

### 3.3. Validation by Enzyme-Linked Immunosorbent Assay (ELISA)

**Figure 3:**
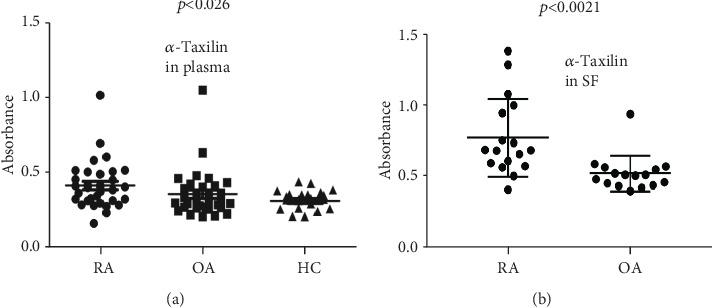
Validation by Elisa. ELISA was performed using plasma (RA = 100, OA = 100, and HC = 64) and SF (RASF = 16 and OASF = 16) sample using anti-*α*-Taxilin antibody followed by HRP-conjugated secondary antibody. Graph showing mean absorbance of ELISA at 492 nm. (a) Graph showing the *α*-Taxilin level in RA plasma sample compared to OA plasma sample and healthy controls (HC). (b) Graph showing the *α*-Taxilin level in SF sample of RA compared to OA.

The ELISA results revealed 1.31-fold and 1.21-fold upregulation of *α*-Taxilin expression in RA plasma compared to HC and OA plasma, respectively (*p* ≤ 0.026). Further, the ELISA results of RASF revealed 1.50-fold higher expression of *α*-Taxilin in RA compared to OA with significant a *p* value (*p* ≤ 0.0021) (Figure 3(b)). However, we do not found a much significant difference of *α*-Taxilin expression between OA plasma and HC plasma (Figure 3(a)) which supports specificity of *α*-Taxilin for RA.

### 3.4. Validation by Fluorescence-Activated Cell Sorting (FACS)

**Figure 4:**
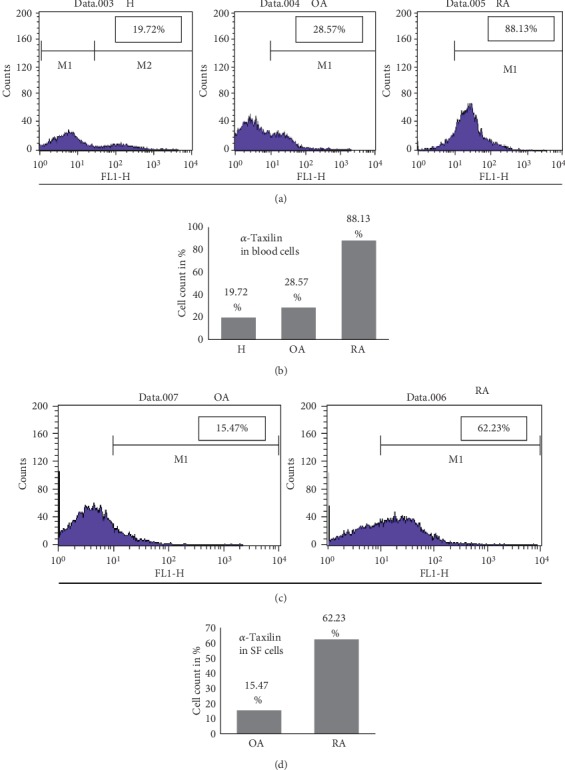
Validation by FACS. Blood cells or SF cells from RA, OA, and healthy blood cells were isolated and proceeded for FACS analysis with *α*-Taxilin primary antibody followed by Alexa Fluor 467-tagged secondary antibody. (a) Data captured using FACS caliber showing percentage of cells positively stained with Alexa Fluor 467 in H, OA, and RA blood cells. (b) Graphical representation of FACS data in terms of cells stained with Alexa Fluor for H, OA, and RA samples. (c) Data captured using FACS caliber showing the percentage of cells positively stained with Alexa Fluor 467 in SF cells. (d) Graphical representation of FACS data in terms of cells stained with Alexa Fluor for OA and RA SF cells.

There were 88.13% positively stained leukocytes with *α*-Taxilin in RA as compared to 28.57% in OA and 19.7% in HC (Figures 4(a) and 4(b)). The expression of *α*-Taxilin in RA blood cells was 4.4 and 3.0 times higher as compared to HC and OA, respectively, whereas the expression of *α*-Taxilin was found to be 1.4 times higher in OA as compared to HC.

FACS analysis on synovial fluid cells was found to follow similar trends as observed in FACS performed on leukocytes. The data revealed that the total percentage of positively stained SF cells with *α*-Taxilin in RA was 62.3% and in OA was 15.4% (Figure 4(c)), suggesting 4 times higher expression of *α*-Taxilin in RASF cells compared to OASF (Figure 4(d)).

### 3.5. Validation by Immunohistochemistry (IHC) and Real-Time PCR

**Figure 5:**
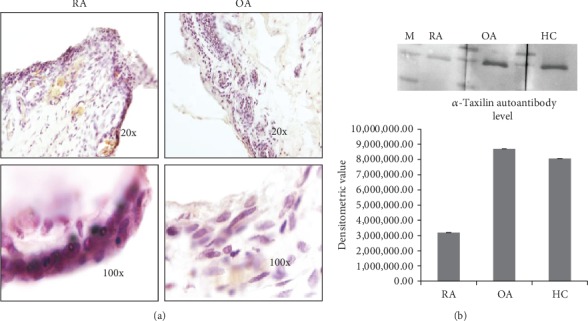
Expression of *α*-Taxilin in the synovium. (a) Tissue section were cut and stained with anti *α*-Taxilin primary antibody followed by HRP-tagged secondary antibody and subsequently developed by DAB then an image taken at 20x and 100x shows high expression of *α*-Taxilin protein in the synovium of RA as compared to OA and healthy control. Pink color represents the level of *α*-Taxilin in the synovium sample. (b) Comparative densitometric analysis of Western blot of the pool sample showing the level of circulating autoantibody against *α*-Taxilin in RA, OA, and healthy plasma.

The expression of *α*-Taxilin was further checked in the synovium by the IHC method. Higher expression of *α*-Taxilin was observed in RA synovium at pannus formation as compared to a section of OA synovium where no pannus formation was observed (Figure 5(a)). The results were validated at the mRNA level by real-time PCR (data not shown).

### 3.6. Autoantibody Detection

The level of circulating autoantibody against *α*-Taxilin in the RA, OA, and HC plasma was analysed. The autoantibody level present in RA against *α*-Taxilin is much lower than that in HC and OA; however, there is no significant increase in OA compared to HC. The expression level of autoantibody in RA patients has been found to be significantly downregulated (0.396-fold downregulated) compared to OA and HC (Figure 5(b)).

### 3.7. String Pathway Analysis and Interaction Study

**Figure 6:**
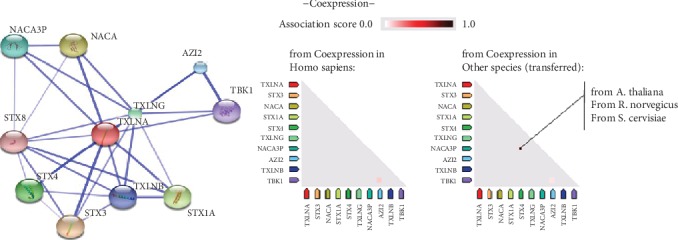
“STRING” pathway analysis and interaction study. In silico study of interacting partner of *α*-Taxilin protein using string pathway analysis. The binding affinity of the proteins is shown in Homo sapiens as well as in other species in terms of the association score.

The *in silico* pathway analysis was carried out by protein-protein analysis online tool “STRING” and revealed that *α*-Taxilin is interacting with ten proteins. The results from the association score of proteins showed that *α*-Taxilin is strongly associated with five proteins (Figure 6), namely, “Nascent polypeptide associated complex subunit alpha” (NACA), syntaxin-4 (STX4), syntaxin-3 (STX3), syntaxin 1A (STX1A), and Gamma Taxilin (TXLNG).

## 4. Discussion

Proteomics is a promising approach in identifying the disease-associated proteins. We have focused our study to find out the differentially expressed proteins in RA using proteomic approach and identified 7 differentially expressed proteins from RASF cells (Table 1). Among these identified proteins, 6 proteins: regulatory protein E2, shikimate kinase, *α*-Taxilin, protein kinase A type 1a regulatory subunit, recombinant signal binding protein, and putative enoyl were found upregulated, while chain A structure of Hop Tpr2a domain protein was observed to be downregulated in RASF cells compared to OASF cells. Regulatory protein E2 plays a role in the initiation of viral DNA replication and observed to be downregulated in aged smokers and patients with chronic obstructive pulmonary disease [28]. Shikimate kinase catalyzes the specific phosphorylation of the 3-hydroxyl group of shikimic acid present in the shikimate pathway in bacteria and is essential for the survival of the tubercle bacillus [29]. Downregulation of protein kinase A regulatory subunit was found to be responsible for endocrine and other tumors [30]. Putative enoyl reductase has a role in very long-chain fatty acid metabolic process, and chain A structure of Hop Tpr2a domain has role in protein-protein interactions [31]. Since RA is an autoimmune disease, we found that two proteins, namely, “*α*-Taxilin” and “recombinant signal binding protein” directly linked with autoimmune response. Both of these proteins have a role in B-cell differentiation, immune responses, and interleukin secretion [21]. Upregulated levels of *α*-Taxilin have been observed in renal cell carcinoma [19] and in development of autoimmunity in IL-14*α*-transgenic mice [21]. It also participates in the transferrin receptor recycling pathway by interacting with sorting nexin 4, involved in cytokine activity [20], B-cell activation, and exocytosis [21]. We also have found that *α*-Taxilin is one of the significant upregulated proteins in RA in our preliminary screening and moreover, its role in RA is yet not explored.

The level of *α*-Taxilin was also found upregulated in other diseases also such as in hepatocellular carcinomas, renal cell carcinoma, and astrocytic tumor and in proliferating neural stem cells during embryonic development in rat [32, 33]. Taxilin interact with the syntaxin family which is well known for their role in intracellular membrane fusion and regulation of exocytosis. It has been seen that the inflammatory marker has correlation with cancer [34]. Moreover, remodeling of tissues takes place in both RA and cancer [35, 36]. However, it is not clear to date whether Alpha-Taxilin takes part in tissue remodeling or not. It may be possible that Taxlin may act as a common pathway regulator for inflammation and cancer response.

In order to strengthen our finding and to find out the role of *α*-Taxilin in the pathogenesis of RA, validation assays were extensively carried out by Western blot and ELISA. SF cells, synovium, SF, and plasma sample were checked for the presence of *α*-Taxilin and has been found upregulated in RA compared to their respective controls. This was the first report where upregulation of *α*-Taxilin has been seen in RA plasma, RASF cell, and RASF. After analyzing the Western blot, we found a higher (3.6-fold and 1.52-fold) expression of *α*-Taxilin in RASF and RAPL as compared to OASF and HC plasma, respectively, while the results from ELISA revealed that there is 1.5-fold and 1.31-fold increase in the *α*-Taxilin level in RASF and RAPL, respectively. This was further strengthened by FACS analysis where we found a nearly 4-fold increased level in RAPL compared to OA and HC (Figure 4(b)). The significant upregulated expression of *α*-Taxilin in FACS along with ELISA from SF (Figure 3(b)) revealed that 89% of RA patients gave positive expression of *α*-Taxilin compared to OA and HC. The results were therefore further strengthened by IHC results using the synovium. The higher expression of *α*-Taxilin at pannus formation indicated its involvement in the pathogenesis of RA compared to OA. As per the literature, specificity of anti-CCP and RF factor was found to be 50% and 40%, respectively; however, the specificity was found 96% and 55%, respectively [37]. The cytokines have many limitations in RA diagnosis due to the effect of age, gender, and diet, and variability [38] thus has not been evaluated. We have compared anti-CCP and RF factor with the current marker in our biological sample. The sensitivity and specificity for Alpha-Taxilin has been found to be 79% and 50%, respectively, compared to RF and Anti-CCP. Though the sensitivity of Taxilin is higher than both the anti-CCP and RF, the specificity remains below that of the anti-CCP. Thus, addition of the Alpha-Taxilin level can be helpful for the evaluation of RA diagnosis and pathogenicity. It is reported that overexpression of *α*-Taxilin induces autoimmunity in transgenic mice [21] where IFN-*γ* was observed to show initiation of autoimmune response. Similarly, T-cell cytokines such as IL-2 and IFN-*γ* were also found to be involved in RA pathogenesis [3, 39]. The activation of B cell by Taxilin leads to secretion of inflammatory cytokines. The pathway followed by Taxilin to induce inflammation is not clearly understood, but IFN-*γ* may be one of the key factors involved for initiation of autoimmune response. Report shows that the IFN-*γ* level has a correlation with Alpha-Taxilin upregulation in a mouse model [21]. As RA is an autoimmune disease, findings of similar biological disease-associated activity in the progression of autoimmunity in the development of RA, indicating that *α*-Taxilin may be one of the key role players responsible for the onset of RA.

To get further insight on the interacting partner involved in signaling pathways with *α*-Taxilin, *in silico* interaction study was carried out. The study revealed five interacting partners such as NACA, STX4, STX3, STX1A, TXLNG. Binding of *α*-Taxilin with the syntaxin family (STX4, STX3, and STX1A) is reported to play a primary role in the regulation of vesicle exocytosis and cytokine-mediated signaling pathway and regulation of immunoglobulin. An IgE secretion implicates its possible role in pathogenesis of RA. The “NACA” also interact with *α*-Taxilin that is known to have a preventive role in appropriate targeting of nonsecretary polypeptide and regulation of cell proliferation and is responsible for muscle fiber development [40]. Furthermore, interestingly, we found a downregulation (0.396-fold, Figure 5(b)) of autoantibody levels in RA plasma compared to HC. The decreased levels of autoantibody thus drew our attention towards the Paul Eherlich's statement about the existence of anti-autotoxin antibodies. It is reported that the decreased level of anti-autotoxin antibodies may lead to the disturbance of homeostasis [41]. Studies have also shown that the central nervous system (CNS) of trauma spontaneously evokes a beneficial T cell-dependent immune response that reduces neuronal loss [41] indicating that the presence of autoantibody has an important role in maintaining homeostasis and protection against collateral damage due to a disease. Further studies are needed to be done to know whether the upregulation of *α*-Taxilin is the result of the failure in subsequent anti-autotoxin autoantibody production.

## 5. Conclusion

Our study therefore concludes that *α*-Taxilin might be one of the prerequisite factors responsible for the onset of immune response in RA while considering our finding of significantly upregulated expression of *α*-Taxilin in RA against HC and OA. The study will be helpful towards the development of a novel prognostic marker to understand further understanding for the development of autoimmune response in RA.

## Figures and Tables

**Table 1:** List of identified differentially expressed protein-proteins.

S. no.	Spot	Protein name	Accession no.	Peptide match	Score	Prime function
1	1	Regulatory protein E2	VE2_HPV58	7	54	Plays a role in the initiation of viral DNA replication,
2	2	Shikimate kinase	AROK_PELLD	8	56	Catalyzes the specific phosphorylation of the 3-hydroxyl group of shikimic acid
3	3	TXLNA protein (Homo sapiens)	Gi|28302156	4	45	Cytokine activity, B-cell activation, and exocytocis
4	4	Chain A, structure of hop Tpr2a domain in complex	Gi|251836914	7	59	Stress-induced phosphoprotein
5	5	Protein kinase A type 1a regulatory subunit	Gi|15705885	3	38	cAMP-dependent protein kinase regulator activity
6	6	Recombination signal binding protein	Gi|190953	3	34	DNA and RNA binding, B-cell differentiation, angiogenesis, humoral immune responses, IL-4 secretion, etc.
7	15	Putative enoyl reductase	YN67_SCHPO	7	61	Fatty acid elongation, very long-chain fatty acid metabolic process

## Data Availability

Data supporting the findings of this study are available from the corresponding author [Dr. Sagarika Biswas] on request.

## Supplementary Materials

Supplementary Materials Supplementary table 1: summary of the clinical measures of 200 patients (RA = 100, OA = 100) and 64 healthy control people.

## References

[1] Smolen J. S., Aletaha D., Koeller M., Weisman M. H., Emery P. (2007). New therapies for treatment of rheumatoid arthritis. *The Lancet*.

[2] Gonzalez A., Maradit Kremers H., Crowson C. S. (2007). The widening mortality gap between rheumatoid arthritis patients and the general population. *Arthritis and Rheumatism*.

[3] Firestein G. S. (2003). Evolving concepts of rheumatoid arthritis. *Nature*.

[4] Biswas S., Sharma S., Saroha A. (2013). Identification of novel autoantigen in the synovial fluid of rheumatoid arthritis patients using an immunoproteomics approach. *PLoS One*.

[5] Korganow A. S., Ji H., Mangialaio S. (1999). From systemic T cell self-reactivity to organ-specific autoimmune disease via immunoglobulins. *Immunity*.

[6] Fong Y., Tracey K. J., Moldawer L. L. (1989). Antibodies to cachectin/tumor necrosis factor reduce interleukin 1 beta and interleukin 6 appearance during lethal bacteremia. *Journal of Experimental Medicine*.

[7] Aletaha D., Smolen J. S. (2018). Diagnosis and management of rheumatoid arthritis. *Journal of the American Medical Association*.

[8] Zvaifler N. J. (1973). The immunopathology of joint inflammation in rheumatoid arthritis. *Advances in Immunology*.

[9] Kinloch A., Tatzer V., Wait R. (2005). Identification of citrullinated *α*-enolase as a candidate autoantigen in rheumatoid arthritis. *Arthritis Research & Therapy*.

[10] Wolfe F., Mitchell D. M., Sibley J. T. (1994). The mortality of rheumatoid arthritis. *Arthritis and Rheumatism*.

[11] Barhamain A., Magliah R., Shaheen M. (2017). The journey of rheumatoid arthritis patients: a review of reported lag times from the onset of symptoms. *Open Access Rheumatology: Research and Reviews*.

[12] Hitchon C. A., el-Gabalawy H. S. (2011). The synovium in rheumatoid arthritis. *The Open Rheumatology Journal*.

[13] Wang J., Zhou N., Xu B. (2012). Identification and cluster analysis of streptococcus pyogenes by MALDI-TOF mass spectrometry. *PLoS One*.

[14] Brandt K. D., Dieppe P., Radin E. L. (2008). Etiopathogenesis of osteoarthritis. *Rheumatic Disease Clinics of North America*.

[15] Park M., Pyun J. C., Akter H., Nguyen B. T., Kang M. J. (2015). Evaluation of a specific diagnostic marker for rheumatoid arthritis based on cyclic citrullinated peptide. *Journal of Pharmaceutical and Biomedical Analysis*.

[16] Nogami S., Satoh S., Tanaka-Nakadate S. (2004). Identification and characterization of taxilin isoforms. *Biochemical and Biophysical Research Communications*.

[17] Uyeda A., Fukui I., Fujimori K. (2000). MDP77: a novel neurite-outgrowth-promoting protein predominantly expressed in chick muscles. *Biochemical and Biophysical Research Communications*.

[18] Nogami S., Satoh S., Nakano M. (2003). Taxilin; a novel syntaxin-binding protein that is involved in Ca^2+^-dependent exocytosis in neuroendocrine cells. *Genes to Cells*.

[19] Mashidori T., Shirataki H., Kamai T., Nakamura F., Yoshida K. I. (2011). Increased alpha-taxilin protein expression is associated with the metastatic and invasive potential of renal cell cancer. *Biomedical Research*.

[20] Sakane H., Horii Y., Nogami S., Kawano Y., Kaneko-Kawano T., Shirataki H. (2014). *α*-taxilin interacts with sorting nexin 4 and participates in the recycling pathway of transferrin receptor. *PLoS One*.

[21] Shen L., Zhang C., Wang T. (2006). Development of autoimmunity in IL-14*α* transgenic mice. *The Journal of Immunology*.

[22] Kruger N. J. (1994). The Bradford method for protein quantitation. *Methods in Molecular Biology*.

[23] Hussain S., Dutta A., Sarkar A., Singh A., Gupta M. L., Biswas S. (2017). Proteomic analysis of irradiated lung tissue of mice using gel-based proteomic approach. *International Journal of Radiation Biology*.

[24] Tobón G. J., Youinou P., Saraux A. (2010). The environment, geo-epidemiology, and autoimmune disease: rheumatoid arthritis. *Journal of Autoimmunity*.

[25] Hueber W., Tomooka B. H., Batliwalla F. (2009). Blood autoantibody and cytokine profiles predict response to anti-tumor necrosis factor therapy in rheumatoid arthritis. *Arthritis Research & Therapy*.

[26] Ahmad T., Mukherjee S., Pattnaik B. (2014). Miro1 regulates intercellular mitochondrial transport & enhances mesenchymal stem cell rescue efficacy. *The EMBO Journal*.

[27] Szklarczyk D., Morris J. H., Cook H. (2017). The STRING database in 2017: quality-controlled protein–protein association networks, made broadly accessible. *Nucleic Acids Research*.

[28] Suzuki M., Betsuyaku T., Ito Y. (2008). Down-regulated NF-E2 related factor 2 in pulmonary macrophages of aged smokers and patients with chronic obstructive pulmonary disease. *American Journal of Respiratory Cell and Molecular Biology*.

[29] Simithy J., Reeve N., Hobrath J. V., Reynolds R. C., Calderón A. I. (2014). Identification of shikimate kinase inhibitors among anti-*Mycobacterium tuberculosis* compounds by LC-MS. *Tuberculosis*.

[30] Griffin K. J., Kirschner L. S., Matyakhina L. (2004). Down-regulation of regulatory subunit type 1A of protein kinase a leads to endocrine and other tumors. *Cancer Research*.

[31] Carrigan P. E., Sikkink L. A., Smith D. F., Ramirez-Alvarado M. (2006). Domain: domain interactions within Hop, the Hsp70/Hsp90 organizing protein, are required for protein stability and structure. *Protein Science*.

[32] Sakakibara S., Nakadate K., Tanaka-Nakadate S. (2008). Developmental and spatial expression pattern of *α*-Taxilin in the rat central nervous system. *The Journal of Comparative Neurology*.

[33] Ohtoma N., Tomiya T., Tanoue Y. (2010). Expression of *α*-taxilin in hepatocellular carcinoma correlates with growth activity and malignant potential of the tumor. *International Journal of Oncology*.

[34] Il'yasova D., Colbert L. H., Harris T. B. (2005). Circulating levels of inflammatory markers and cancer risk in the health aging and body composition cohort. *Cancer Epidemiology, Biomarkers & Prevention*.

[35] Goldring S. (2005). The effects of inflammatory arthritis on bone remodeling. *Arthritis Research & Therapy*.

[36] Johnsen M., Lund L. R., Rømer J., Almholt K., Danø K. (1998). Cancer invasion and tissue remodeling: common themes in proteolytic matrix degradation. *Current Opinion in Cell Biology*.

[37] Jansen A. L., van der Horst-Bruinsma I., van Schaardenburg D., van de Stadt R. J., de Koning M. H., Dijkmans B. A. (2002). Rheumatoid factor and antibodies to cyclic citrullinated peptide differentiate rheumatoid arthritis from undifferentiated polyarthritis in patients with early arthritis. *The Journal of Rheumatology*.

[38] Burska A., Boissinot M., Ponchel F. (2014). Cytokines as biomarkers in rheumatoid arthritis. *Mediators of Inflammation*.

[39] Zhang J. M., An J. (2007). Cytokines, inflammation and pain. *International Anesthesiology Clinics*.

[40] Rahman A., DeCourcey J., Larbi N. B., Loughran S. T., Walls D., Loscher C. E. (2013). Syntaxin-4 is essential for IgE secretion by plasma cells. *Biochemical and Biophysical Research Communications*.

[41] Shoenfeld Y., Toubi E. (2005). Protective autoantibodies: role in homeostasis, clinical importance, and therapeutic potential. *Arthritis and Rheumatism*.

