# Supplementary material for: Synovial Fluid Cell Proteomic Analysis Identifies Upregulation of Alpha-Taxilin Proteins in Rheumatoid Arthritis: A Potential Prognostic Marker
Source: J Immunol Res. 2020 Apr 23;2020:4897983. doi: 10.1155/2020/4897983 (PMC7195675; doi:10.1155/2020/4897983)
Supplement: Supplementary Materials — Supplementary table 1: summary of the clinical measures of 200 patients (RA = 100, OA = 100) and 64 healthy control people. [file 4897983.f1.docx]

**Supplementary table: 1**

| Summary of the clinical measures of 200 patients (RA=100, OA=100) and 64 Healthy Control people. | | | | |
| --- | --- | --- | --- | --- |
| S. No | characteristics | RA(n=100) | OA(n=100) | HC(n=64) |
| 1 | Mean Age(in Years) ±SD | 50 ± 5 | 50 ± 5 | 45 ± 10 |
| 2 | Female (%) | 80% | 78% | 68% |
| 3 | Mean ESR ±SD | 35 ± 5 | 15 ± 5 | - |
| 4 | RF | +ve | ­ ve | - ve |
| 5 | Mean CRP(mg/lit) ±SD | 80 ±15 | 30 ±10 | 10 ± 5 |
| 6 | Mean Tender Joint ±SD | 20±6 | 3 ± 1 | - |
| 7 | Mean Swollen joint ±SD | 10±4 | 2 ± 1 | - |
| 8 | Mean DAS-28 score ±SD | 6 ± 0.5 | - | - |
| 9 | Mean health score ±SD | 40±10 | 20 ± 10 | 0 ± 5 |
| 10 | Mean disease duration(years) ±SD | 10 ± 5 | 6 ± 3 | - |
| 11 | Medication(Yes/No) | Yes | Yes | No |
